# Lights, Camera…Citizen Science: Assessing the Effectiveness of Smartphone-Based Video Training in Invasive Plant Identification

**DOI:** 10.1371/journal.pone.0111433

**Published:** 2014-11-05

**Authors:** Jared Starr, Charles M. Schweik, Nathan Bush, Lena Fletcher, Jack Finn, Jennifer Fish, Charles T. Bargeron

**Affiliations:** 1 Department of Environmental Conservation, University of Massachusetts, Amherst, Massachusetts, United States of America; 2 Service Forestry, Massachusetts Department of Conservation and Recreation, Amherst, Massachusetts, United States of America; 3 Invasive Species and Information Technology, Center for Invasive Species & Ecosystem Health, University of Georgia, Tifton, Georgia, United States of America; University of Kent, United Kingdom

## Abstract

The rapid growth and increasing popularity of smartphone technology is putting sophisticated data-collection tools in the hands of more and more citizens. This has exciting implications for the expanding field of citizen science. With smartphone-based applications (apps), it is now increasingly practical to remotely acquire high quality citizen-submitted data at a fraction of the cost of a traditional study. Yet, one impediment to citizen science projects is the question of how to train participants. The traditional “in-person” training model, while effective, can be cost prohibitive as the spatial scale of a project increases. To explore possible solutions, we analyze three training models: 1) in-person, 2) app-based video, and 3) app-based text/images in the context of invasive plant identification in Massachusetts. Encouragingly, we find that participants who received video training were as successful at invasive plant identification as those trained in-person, while those receiving just text/images were less successful. This finding has implications for a variety of citizen science projects that need alternative methods to effectively train participants when in-person training is impractical.

## Introduction

Citizen science, the crowdsourcing of scientific data collection by volunteers, is a research model that allows for large-scale data collection that would be otherwise cost-prohibitive [1]–[3]. As a mutually beneficial endeavor, scientists gain data and connect to a community of people interested in their work, while participants gain an opportunity to learn, connect to others with shared interests, and participate in the scientific process. While, ecological citizen science projects have existed in the United States for more than a century [4], technological advances have fuelled rapid expansion in this field over the last decade [4]–[6]. Today, the proliferation of the Internet and the increasing popularity of smartphone technology mean that citizens have access to sophisticated data collection and submission tools on an unprecedented scale [15].

Scientists are increasingly interested in how to leverage these technologies to engage citizens in a growing array of data collection efforts. From projects aimed at monitoring birds, bees, crabs, and snails to those targeting plants, fish, reptiles, fungi, and mammals the list of ecological citizen science projects is growing [5]. Smartphone-based citizen science applications (apps) are a particularly interesting development as they allow citizens to submit photos, video, audio, field notes, and GPS positioning data with the click of a button.

Yet as technology has allowed researchers and volunteers to connect across previously forbidding distances and submit ever more sophisticated types of data, geography can pose a challenge if the participants require training in order to collect the needed data. While the traditional “in-person” training model is quite effective, it can quickly become cost prohibitive as the spatial scale of a project increases or if the researcher is located a great distance away from the area under study. Thus, novel training methods that can be remotely administered are needed and indeed have been employed by various projects.

Some projects, for example, now use online training modules that utilize text, images, games, and video. As Booney and colleagues point out, “Projects demanding high skill levels from participants can be successfully developed, but they require significant participant training and support materials such as training videos” [7]. Yet, while some of these remotely administered online training technologies go back more than a decade, there has been only limited study on the effectiveness of such different training methods. Knowing which training types are most effectual is crucial to successfully designing programs that can acquire high quality data at a reasonable cost.

One study that is particularly relevant to this question is from Newman et al. [8]. They investigate the ability of online static (text) and multimedia (audio-visual) tools to train volunteers to correctly identify invasive plants. They do not include in-person training in their experiment, instead comparing volunteers to professionals. While ‘professionals’ is not explicitly defined, we assume it means people whose job involves working outdoors, in some capacity, with plants. They find professionals are able to correctly identify invasive plants more often than volunteers and that there is no difference in volunteer's effectiveness between the static and multimedia training. Since they do not analyze in-person training however, there is a gap in knowing how volunteers trained with text or video compare to the those trained with the more costly, but tried and true in-person training method. Indeed, in their discussion they call for more research on this comparison.

To address this gap, we analyze three training models. In one scenario we call “Cohort 1: In-person training”, participants are provided in-person training along with app-based videos and app-based text/images. In the second scenario, what we call “Cohort 2: Video-training”, participants are given no in-person training, but receive app-based video and app-based text/image training. In the third scenario, what we call the “Cohort 3: Text/Image only training”, participants only receive app-based text/image training (no video or in-person training). We hypothesized that Cohort 1 (in-person training) would be the most successful at invasive plant identification, followed by Cohort 2 (video-training), and finally Cohort 3 (text/image).

### Outsmart Invasive Species Project

**Figure 1:**
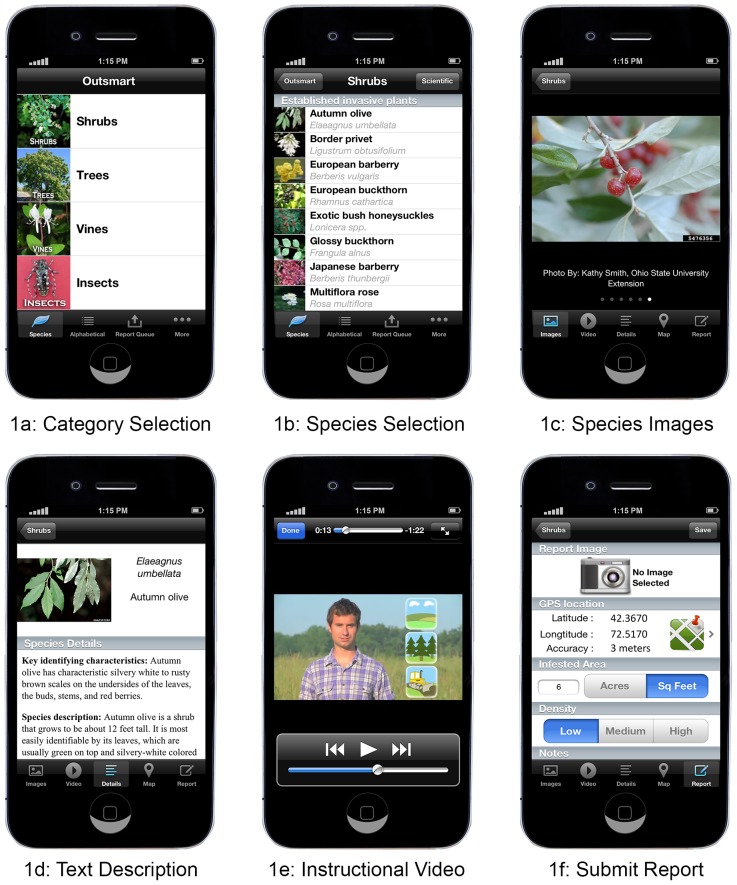
Sample screenshot images from the Outsmart App.

We conducted our experimental study in the context of the Outsmart Invasive Species Project (Outsmart). Outsmart is a collaboration between the University of Massachusetts Amherst, the Massachusetts Department of Conservation and Recreation (MA DCR) and the Center for Invasive Species and Ecosystem Health at the University of Georgia. The project aims to strengthen ongoing invasive species monitoring efforts by enlisting help from citizens across New England (Connecticut, Rhode Island, Massachusetts, Vermont, New Hampshire, and Maine), with a particular focus on Massachusetts. Volunteers are asked to identify and report data on invasive plants and insects in their own time and submit data via a free account through the Early Detection and Distribution Mapping System (EDDMapS) website (www.eddmaps.org) or through our smartphone app called “Outsmart Invasive Species” (“Outsmart” for short; http://masswoods.net/outsmart) (Figure 1). The project leverages the increasing number of people equipped with smartphones or digital camera/web technology and aims to expand the scope of invasive monitoring with a particular focus on early detection of new or emergent threats.

Invasive species monitoring has been identified as a key realm where citizen science can be employed particularly effectively [5]. Invasive species can wreak ecological and economic destruction and once established may be impractical or impossible to eradicate [9]. Thus, as researchers have identified “Monitoring programs aimed at detecting low-density ‘founder’ populations can play a critical role in slowing or even stopping the spread of harmful invasive species by identifying recently established populations that can be targeted for control and/or eradication” ([10], citing [9]). Yet, like other citizen science programs, one hurdle that the Outsmart project faced was how to train participants distributed across New England. To test the effectiveness of remote training, we developed in-person training sessions, training videos, and text/images and conducted a study asking participants to identify five invasive plant species in Massachusetts over the course of the late summer and early fall 2013.

## Methods

### Text Development and Video Production

We began our study by creating a set of training materials. For each species, we developed text and selected pictures that highlighted key features, working with a regional expert, Ted Elliman from the New England Wildflower Society (newenglandwild.org), and imagery available from the University of Georgia's Bugwood Image Database System (http://images.bugwood.org/). The text described distinct characteristics, seasonal changes in appearance, potential look-alikes, and the ecological threat posed by the species. To eliminate potential error from using different language for the different training groups, we aimed to keep the in-person, video, and text scripts as consistent as possible.

We did, however, allow for slight variations to create a smoother presentation of information as text, video, and in-person presentation are fundamentally different mediums. The text version for the Outsmart app contained key characteristics at the top of the page followed by species description, seasonal developments, look-alikes, and ecological threat. The app displays pictures that appear at the top of the smartphone screen with scrolling capability. The user can also click on a picture to zoom-in and pan around on a specific key feature. The text allows users to move around the page at their leisure and easily see the different sections.

The invasive id training videos for use on the app or on the web present information more linearly than text, with less ability to quickly jump to a particular section. In the videos, we began with a shortened description of the ecological threat, used the same language for the key characteristics, used a shortened version of seasonal changes, and used a slightly shorter text for potential look-alikes. The shorter text was deemed to be appropriate because video let us simultaneously present an image while verbally describing it, which reduced the need for a lengthy description. The shorter format was also deemed beneficial as it helped reduce smartphone battery drawdown, which is important if users view the videos while in the field.

The script used for in-person training was based on the video scripts and the instructor was careful not to offer additional special hints or clues. Participants of the in-person sessions were allowed to ask questions, which meant they could receive some additional information.

While aware that any differences in information given to the text/image, video, and in-person groups created variability, we concluded that slight variations were appropriate as they best captured real world applications of the three training methods. Therefore, by keeping training relatively standardized while allowing for the inherent strengths of the three methods to be captured, we reached a design representative of actual application in the field.

### Volunteer Recruitment

During the summer of 2013 we worked with two interns funded by The Nature Conservancy to recruit participants at fifteen events (e.g., festivals, fairs, etc.) throughout the region. We also posted recruitment materials on the Outsmart project website and emailed anyone who submitted an invasive species report after July 1 to encourage them to participate in the study. Additionally, students in three Natural Resource Conservation courses at the University of Massachusetts Amherst were recruited to volunteer. All volunteers were directed to complete the online survey in order to participate in the study.

### Survey

The survey was designed using Survey Gizmo, a web-based survey design tool (surveygizmo.com) and was pre-tested with colleagues and students. To increase the number of respondents the survey included institutional icons at the top for legitimacy, length of time the survey would take, end-date of the experiment to create urgency, research context to emphasize significance and importance, and disclosures that data would be kept private.

In the survey, participants were asked to self-identify their plant ID experience level as “No experience”, “Beginner”, “Intermediate”, or “Advanced”. Participants who identified as “Beginner” “Intermediate” or “Advanced” were asked an additional two questions. The first question asked them to identify how they learned their plant ID skills. The second asked if they could specifically identify, without aids, the five species used in the experiment (described in Study Design section below). In addition, the survey collected data on gender, age, and education level.

Upon completing the survey, participants were randomly assigned, via an automated process in Survey Gizmo, to text/image, video, or in-person training. Depending on the cohort they were assigned to, participants received different instructions upon completing their survey. All instructions included links to both the Android and iPhone Outsmart application, links to create an EDDMapS account (a prerequisite for using the Outsmart mobile app), instructions on using the app, and contact information for the research team. The instructions for Cohort 1 (in-person training) told participants they would have to attend an in-person training session at one of three established times and they were also encouraged to use embedded text/images and video to identify the five target species. If participants in this group were unable to attend one of the training sessions they were re-assigned to the text/image or video group. The instructions for Cohort 2 participants (app with video training) told them that they could use the embedded training videos in addition to the embedded text/images to identify the five target species. The instructions for Cohort 3 (text/image training only) told participants to use only the app-embedded text/images to identify the five target species.

### Study Area

While the Outsmart project has participants across New England, for the purposes of the experiment we limited our study to Massachusetts. Massachusetts is the eighth most forested state in the nation and has a wide variety of topography and forest types ranging from mountainous spruce-fir-northern hardwoods in the western portion of the state to the coastal plains and lowland pitch pine-scrub oak forests to the east [11]. Massachusetts was also one of the first states to be settled by Europeans and has undergone extensive land-use change since that time [12]. These factors, in combination with accidental and intentional introductions of non-native species led to the occurrence of many different species of invasive plants in the state. Massachusetts Division of Fisheries and Wildlife's Natural Heritage Endangered Species Program in partnership with the Massachusetts Invasive Plant Advisory Group (MIPAG) have identified 67 plant species that are either "Invasive," "Likely Invasive," or "Potentially Invasive" [13].

### Study Design and Data Collection

Of the many invasive species found throughout Massachusetts, we limited our experimental study to five common species that represents invasive plant species both relatively easy and difficult to identify. Our assessment of a species difficulty was based on conversations with field biologists at the U.S. Fish and Wildlife Service, examples in the literature for some species [8], and a survey of University of Massachusetts students in a plant identification course. The easy to identify species were: Japanese knotweed (*Fallopia japonica*), autumn olive (*Eleagnus umbellata*), and multiflora rose (*Rosa multiflora*). The more difficult species were: glossy buckthorn (*Alnus frangula*) and exotic honeysuckles (*Lonicera spp*). Honeysuckles are actually a group of species in the same genera, but are referred to as a single species here for simplicity.

The general inspiration for our study design came from our desire to mimic the experience of actual Outsmart users as closely as possible. After receiving training, we asked participants to use the app from June 1, 2013 through September 30, 2013 and report any suspected occurrences of the five assigned species. We deemed this timeframe to be appropriate as it encouraged as many submissions as possible while limiting potential bias due to seasonal change in plant appearance. Just like a regular Outsmart user, participants in the experiment were allowed to report any sightings, whether they specifically went out looking for the invasive species or if they just happened to notice them while out hiking, walking, or doing other outdoor activities. User submissions were verified as correct or incorrect by Outsmart researchers utilizing the EDDMapS interface.

### Data Analysis

We received 534 total submissions from 78 participants: 19 in Cohort 1 (in-person trained), 24 in Cohort 2 (app and video training), and 33 in Cohort 3 (text and image trained group). Although these volunteers were randomly assigned to groups of equal size, the final groups were unbalanced due to participants dropping out of the study. It is likely that the in-person group had the smallest number of participants because of the additional time and effort required to show up for in-person training. One participant was dropped from further analysis because they submitted data from northern Vermont, which was outside our study area of Massachusetts. A second participant was dropped because they only submitted data for non-targeted species for the experiment. One record was dropped from one participant because a picture was not included. We ended up with 529 usable submissions from 76 participants.

When we examined our submissions by date, we discovered that 95% of our data was submitted between September 2 and September 30. This gave us additional confidence that our results would not be biased by seasonal changes in plant appearance. The September-skew may be due to general “participant procrastination” or the fact that students formed a large contingent of our participants. It may be that these students chose to defer their participation in the experiment until they were back at the university.

To analyze the data further, we generated a descriptive statistic for the percent correct, by training type (cohort), for each species of plant. This was generated by aggregating data from all users and then dividing the number of correct submissions for each species by the total number of submissions for that species.

We next analyzed the data at the individual participant-level. For each participant we divided the number of correct submissions by the total number of submissions, which yielded a percent correct score. We then used one-way analysis of variance (ANOVAs) to look for differences across all training types and also between training types [8]. Because the participant's percent correct scores was sensitive to low numbers of submissions we only used data from participants that submitted 5 or more observations when conducting these ANOVAs. This yielded a total of 56 participants: 14 in the in-person training cohort, 17 in the video training cohort, and 25 in the text and image training cohort.

Because our samples included individuals with different plant ID experience levels (beginner, intermediate, and advanced), ages, and education levels we also ran a generalized linear model (GLM) using the raw data on number of correct submissions out of total submissions as our dependent variable [14]. We used a binomial distribution, and weighting the number of submissions so that those who submitted more data were given more weight in the model. This allowed us to confidently use data from all 76 participants without low submission levels skewing the results. We ran a series of GLMs testing for effects and interactions between the plant id experience, training type, age, and education variables. Lastly, to decouple any interactive effects, we ran GLMs for each plant ID experience level (beginner, intermediate, and advanced) to see the effect of training type on percent correct within each levels of experience.

## Results

The percent correct by species shows that for all training types there were reasonably high levels of correct submissions (Table 1). Multiflora Rose, for example, had almost 100% correct submissions in all three training groups. At the other extreme, exotic honeysuckle seemed to be fairly difficult to identify, regardless of training type, although video (60%) and in-person training (57%) had higher levels of correct submissions than text/image (46%). Looking across all species, in-person (cohort 1) and app-based video training (cohort 2) had a higher percent of correct submissions (92%) compared to the text/image trained users (cohort 3; 81%).

**Table 1:** Percent correctly identified by the five species investigated.

ID Difficulty	Species	In-Person (Cohort 1)	Video (Cohort 2)	Text/Images (Cohort 3)
**Easy**	Autumn Olive	76%	86%	84%
	Japanese Knotweed	97%	98%	84%
	Multiflora Rose	98%	96%	98%
**Difficult**	Exotic Honeysuckles	57%	60%	46%
	Glossy Buckthorn	100%	89%	75%
**Total Mean**	*all species*	92%	92%	81%

**Figure 2:**
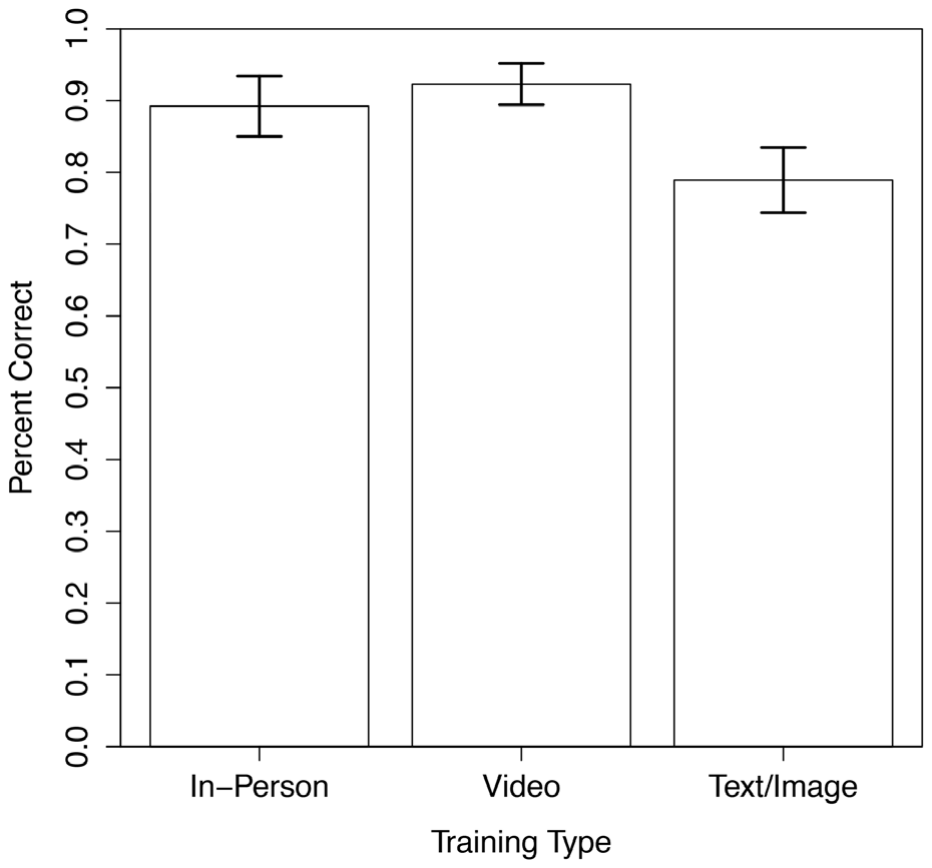
Percent correctly identified by training type.

Looking at percent correct by individual participants, the ANOVA across all training types reveals that training method does play a significant role in influencing ability to correctly identify invasive plants (F = 3.07; p = 0.05; df = 2). Yet, encouragingly, with all three training types that were studied, volunteers did reasonably well at correct identification (Figure 2). Even those who just received text/image training had a mean correct plant identification score of 79%. Those receiving the additional video training had a mean correct ID score of 92%. And those receiving the additional in person training had a mean of 89%. (Note the difference in training type means between Table 1 and Figure 2. The Table 1 mean is calculated by pooling submissions from all users and then dividing the number of correct submissions by the total number of submissions. Whereas the Figure 2 mean is calculated by first assigning a percent correct submissions score to *each user* and then using those scores to calculate a group mean.)

The between group ANOVAs revealed a significant difference between training types. Cohort 2 who received video training did significantly better at plant ID than those in cohort 3 who were taught with just text and images on the app (F = 4.97; p = 0.03; df = 1). Interestingly, although in-person training had a mean correct ID score of 89% compared to 79% for text and image, the ANOVA did not find a statistically significant difference between these training types (F = 2.26; p = 0.14; df = 1). We suspect this is due to high variance from our low sample size of only 14 participants in the in-person cohort 1 group. Finally, we found video training (cohort 2) and in-person training (cohort 1) to be comparable, with no significantly different between these two training groups (F = 0.39; p = 0.54; df = 1).

In the GLMs, age and education were not significant variables and models that included them did not perform significantly better than simpler models, so we chose a GLM that just included an interaction between training type and plant ID experience. This showed a significant interaction between these variables for those in cohort 2 who received video training and had a moderate level of plant ID experience (B = −2.60; SE  = 0.89; p = 0.003).

**Figure 3:**
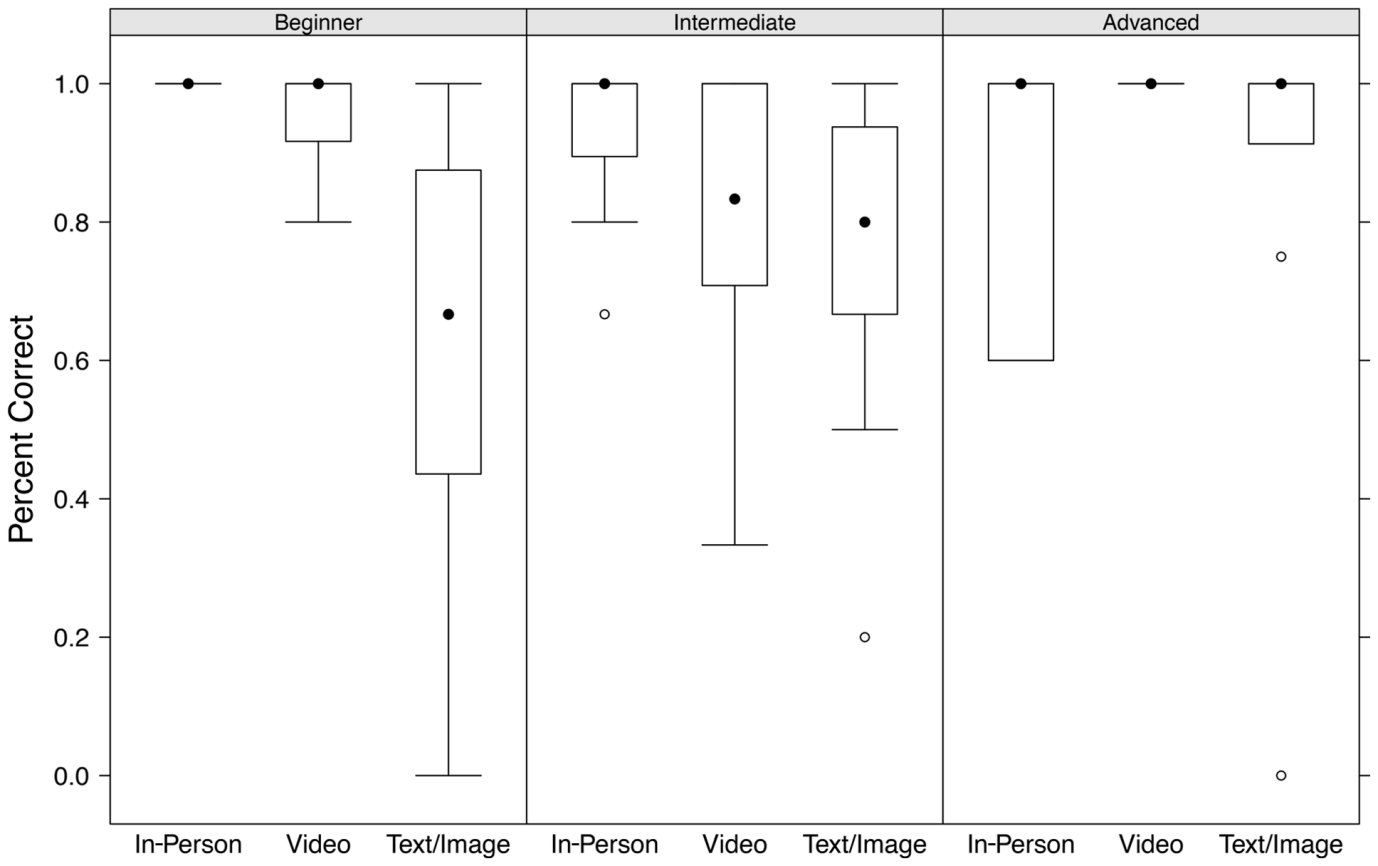
Percent correct by training type and plant ID experience.

To decouple this interaction, we ran GLMs for each level of plant ID experience with percent correctly identified as the dependent variable and training type as the independent variable. For the plant ID beginner group we found those who received video training (cohort 2) did better than those who received text/image training and the difference was highly significant (B = 2.72; SE  = 0.79; p = 0.0006). Those who received in-person training also did significantly better than those who received text/image training, achieving a perfect 100% correct ID rate (Figure 3).

The GLM for the intermediate plant ID experience group found that although those who received video training (cohort 2) had a slightly higher median correct ID rate than those who received text/image training (cohort 3), the difference was not statistically significant (B = 0.12; SE  = 0.41; p = 0.76). Those who received in-person training (cohort 1) however did do significantly better than those receiving text/image training (cohort 3; B = 1.22; SE  = 0.48; p = 0.01) (Figure 3). Finally, the GLM for the advanced or expert plant ID experience group found no significant difference between the three training types. All training type groups had a median correct ID of 100% (Figure 3).

## Discussion

### Plant Species

The percent correct by species shows that in general our understanding of the species' difficulty were reasonably accurate (Table 1). An exception may be Glossy Buckthorn and Autumn Olive. Glossy Buckthorn was classified as difficult, and this seems correct for those who received text/image training, but for those who received video on the app or in-person training Autumn Olive actually had lower percent correct scores. This may be because Russian Olive is quite common and similar in appearance to Autumn Olive, which led to more false reports for this species.

Exotic Honeysuckle, as expected, proved to be the most difficult species to identify across all three training types and speaks to the limitations of effectively training citizen scientists when it comes to very difficult to identify species. Although, the fact that video training was on par with in-person training (in fact doing slightly better for Exotic Honeysuckle identification) suggests that this training method may be as effective as in-person training even for difficult species.

Across all species, we find that all training types were fairly effective with even submissions from the text/image training group being correct 81% of the time (Table 1). Our hypothesis generally held true across species that those with app-based video training would do better than text/image training and in-person training would do the best. Encouragingly though, for the feasibility of broadly providing training to many people, submissions from the video-trained cohort actually outperformed the in-person training cohort in 3 of the 5 plant species. These findings need to be considered carefully though as the interactive effect of previous plant ID experience and training type is not taken into account in these descriptive statistics.

### Training Types

Looking at the average percent correct of individuals gives us additional confidence that the training types were significantly different. Our expectations were met that the text/images participants would have lower correct plant ID scores than the other two training types (Figure 2). Surprisingly, participants trained with video on the app actually had a slightly higher mean percent correct score than those trained in person, but this was not a statistically significant difference. *We expected in-person training to do the best across the board, so this is an exciting finding as it shows that video can be at least as effective for training citizen scientists in plant ID.*

Additionally, even though text/image training via the app was not as effective as app-based video or in-person training, the mean plant ID score was still 79% (Figure 2). This suggests it is possible for citizen science projects to use remote training methods and still acquire high quality data. And if well-produced videos are used for the training, the submitted data may be on par with that submitted by those trained in-person. This has encouraging implications for expanding the scope and scale of citizen science projects.

One caveat for the text/image group is that we did not have a way to restrict their access to the app-embedded training videos. Instead we explicitly instructed them to only use text/images and trusted them to comply. We also did not have a way to prevent participants in any of the groups from using outside resources such as guidebooks or online materials. However, as the results largely confirmed our expectations on training type and plant ID ability, it would appear that participants followed our instructions and did not cheat.

### Plant ID Experience

Looking at the effects of training type, while controlling for previous plant ID experience, are encouraging. The group classified as beginners are those we are most interested in because they had little to no previous plant ID experience. This group allows us to most clearly see the effect of the different training methods. Here we see that those receiving text/image training had a median percent correct score in the mid 60% range, but there was a large variance (Figure 3). The group trained with video on the app was more homogenous, with a median percent correct score of 100%. Finally, those trained in person had a perfect score of 100% correct plant ID. The sample sizes for all three groups were fairly low, but the results are encouraging and suggest there is value in further study.

The effect of training type on the intermediate group was a bit less clear. In-person training was significantly better than app-based text/image, but app-based video was statistically equivalent to app-based text/image training. The video group had a large variance, which we attribute to a small sample size.

Finally, the advanced plant ID experience group had results that we expected. Regardless of training type they all had median plant ID scores of 100% correct (Figure 3). This is logical considering people that already possessed advanced plant ID experience weren′t influenced by training, since they likely already knew how to identify the targeted species. One interesting aspect of this group however is the large variance in the in-person training cohort, where some people that self-identified as “advanced” actually did quite poorly at plant ID. We are calling this phenomena the “overly confident” or “arrogance” factor, meaning it appears some participants incorrectly thought they already knew how to identify targeted plants and failed to pay attention during in-person training, which led to lower correct identifications.

One caveat to this component of the study is that participant's plant ID classification was derived from a self-classification on our survey. We did not independently test or verify their experience. Yet, as the results largely match our expectations for the groups, we believe that this self-classification was a generally accurate reflection of participants' abilities.

### Limitations

While app-based text/image training was quite effective at plant ID and app-based video training was shown to be equivalent to in-person training, it is important to mention the limitations of our study. We only targeted five invasive plant species and our study was confined to the Commonwealth of Massachusetts. It is unclear how transferable these findings may be to other species and geographic areas. While we tried to study a range of difficulty levels, the relative difficulty of Exotic Honeysuckle ID, may suggest that for very difficult species citizen science projects may have trouble training volunteers to correctly identify a species, even with in-person training.

## Conclusion and Recommendations

As app-based video was shown to be an effective tool for remote training, we recommend building regional and national invasive species training video databases. Since the initial study, our project partnered with the Nature Conservancy's Healthy Trees Healthy Cities initiative to produced eight additional training videos focused on invasive insects. All our videos are freely available on YouTube and are available to use for citizen science projects around the country. This and other projects like it could form collaborative networks to create and share video training resources. These types of videos could also be leveraged for instruction in Massive Open Online Courses (MOOCs). The MOOC platform allows instructors to train students remotely and we could easily foresee our videos being used for a plant ID MOOC. As MOOCs continue to develop we see the potential for a variety of partnerships between citizen science projects and online courses, collaborating to produce shared video resources. Like all instructional methods however, it is important to note that to be effective the videos need to be of high quality and be able to engagingly communicate information.

Citizen science can generate vast quantities of data and allow projects to take place that would otherwise be logistically unfeasible or cost-prohibitive to conduct. One limiting factor in these studies is how to effectively train participants in an efficient and inexpensive manner. While in-person training is effective, this option may not be realistic or cost effective for large projects that seek data over a vast spatial scale. Encouragingly, we find that remotely administered training via a smartphone app can be effectively employed to train citizen scientists, with video-based training being generally equivalent to in-person training. While further study with larger samples and more species is needed and while further study within different types of project is encouraged, our study suggests that citizen science projects need not be limited by an inability to effectively train participants remotely. Smartphone-based video training can help.

## References

[1] Cohn J (2008) Citizen science: Can volunteers do real research? BioScience 58(30: 192–197 10.1641/B580303

[2] Lepczyk C, Boyle O, Vargo T, Gould P, Jordan R, et al.. (2009) The increasing acceptance, role, and importance of citizen science in ecology. Symposium reports from the 2008 ESA annual meeting. Bulletin of the Ecological Society of America: 308–317. July.

[3] Nov O, Arazy O, Anderson D (2014) Scientists@Home: What drives the quantity and quality of online citizen science participation? PLoS ONE. 9(4): e90375 10.1371/journal.pone.0090375 PMC3972171 24690612

[4] Greenwood J (2007) Citizens, science and bird conservation. Journal of Orinithology 148 suppl 1 S77–S124 10.1007/s10336-007-0239-9

[5] Dickinson J, Zuckerberg B, Bonter D (2010) Citizen science as an ecological research tool: Challenges and benefits. Annual Review of Ecology, Evolution and Systematics 41: 149–172 10.1146/annurev-ecolsys-102209144636

[6] Teacher A, Griffiths D, Hodgson D, Inger R (2013) Smartphones in ecology and evolution: A guide for the app-rehensive. Ecology and Evolution 3(16): 5268–5278 10.1002/ece3.888 24455154 PMC3892334

[7] Booney R, Cooper C, Dickinson J, Kelling S, Phillips T, et al (2009) Citizen science: A developing tool for expanding science knowledge and scientific literacy. BioScience 59(11): 977–984 10.1525/bio.2009.59.11.9

[8] Newman G, Crall A, Laituri M, Graham J, Stohlgren T, et al (2010) Teaching citizen science skills online: Implications for invasive species training programs. Applied Environmental Education and Communication 9(4): 276–286 10.1080/1533015X.2010.530896

[9] Lodge D, Williams S, MacIsaac H, Hayes K, Leung B, et al (2006) Biological invasions: Recommendations for U.S. policy and management. Ecological Applications 16(6): 2035–2054 10.1890/1051-0761(2006)016 [2035:BIRFUP]2.0.CO;2 17205888

[10] Fitzpatrick M, Preisser E, Ellison A, Elkington J (2009) Observer bias and the detection of low-density populations. Ecological Applications 19(7): 1673–1679 10.1890/09-0265.1 19831062

[11] de la Cretaz A, Fletcher L, Gregory P, VanDoren G, Barten P (2010) An assessment of the forest resources of Massachusetts. University of Massachusetts Amherst and Massachusetts Department of Conservation and Recreation, 274 pp.

[12] Foster D, O′Keefe J (2000) New England forests throughout time: Insights from the Harvard Forest dioramas. Cambridge, MA: Harvard University Press.

[13] Massachusetts Invasive Plant Advisory Group (MIPAG) (2014). http://www.massnrc.org/mipag/. Accessed June 10, 2014.

[14] Zuur A, Leno E, Walker N, Saveliev A, Smith G (2009) Mixed effects models and extensions in ecology with R. Springer, New York.

[15] Bonney S, Phillips T, Wiggins A, Ballard H, Miller-Rushing A, et al (2014) Next steps for citizen science. Science 343(6178): 1436–1437 10.1126/science.1251554. 24675940

